# Reviewer acknowledgement

**DOI:** 10.1186/s12981-016-0097-8

**Published:** 2016-03-04

**Authors:** Eric Arts, Mark Boyd

**Affiliations:** Western University, London, Canada; Kirby Institute for Infection and Immunity in Society, Sydney, Australia

## Contributing reviewers

*AIDS Research and Therapy* is very grateful for the generously-provided time and constructive, insightful reports provided by the journal’s highly-qualified reviewers. We would like to show our appreciation by thanking the following people for their assistance with review of manuscripts for the journal in 2015.

**Kevin Arien**

Belgium

**Tristan Barber**

UK

**Stephen Bar**

Canada

**Edward Berger**

USA

**Javier Bermudez-Silva**

Spain

**Lionel Berthoux**

Canada

**Sanjay Bhagani**

UK

**Andrew Blance**

Australia

**Mark Bloch**

Australia

**David Boettiger**

Australia

**Rohan Bopage**

Australia

**Bruce Brew**

Australia

**Paul U. Cameron**

Australia

**Thomas Campbell**

USA

**Dianne Carey**

Australia

**Kieran Cashin**

Australia

**Derek Chan**

Australia

**Ploenchan Chetchotisakd**

Timor-Leste

**Ting Soo Chow**

Malaysia

**David Clifford**

USA

**Eric Cohen**

Canada

**Lois Conley**

USA

**Suzanne Crowe**

Australia

**Mark Danta**

Australia

**Mark De Souza**

USA

**Greg Dekaban**

Australia

**Mark Douglas**

Australia

**Gwamaka Eliudi**

USA

**Julian Elliot**

Australia

**Jacqueline Flynn**

Australia

**Nathan Ford**

Switzerland

**Martyn French**

Australia

**Yong Gao**

USA

**Roger Garsia**

Australia

**Davide Gibellini**

Italy

**Charles Gilks**

Australia

**Jonathan Golub**

USA

**David Gracey**

Australia

**Michael Grant**

USA

**Marguerite Guiguet**

France

**Manoji Gunathilake**

Australia

**Bridget Haire**

Australia

**Claudia Hawkins**

USA

**C. C. Hung**

Taiwan

**Awachana Jiamsakul**

Australia

**Chandy John**

USA

**Phyllis Kanki**

USA

**Richard Kaplan**

South Africa

**David Katzenstein**

USA

**Phillip Keen**

Australia

**Shane Kelly**

Australia

**Stephen Kerr**

Thailand

**Brian Kigozi**

Uganda

**Amelia Knopf**

USA

**Kersten Koelsch**

Australia

**John Koethe**

USA

**Scott Letendre**

USA

**Patrick Li**

China

**Michaela Lucas**

Australia

**Grace Lui**

Hong Kong

**Patrick Mallon**

Ireland

**Jamie Mann**

Canada

**Weerawat Manosuthi**

Thailand

**Louis Mansky**

USA

**Marianne Martinello**

USA

**Esteban Martinez**

USA

**Paul McKay**

UK

**James McMahon**

Australia

**Sanjay Mehta**

Canada

**D. Mohan**

USA

**Patricia Molina**

USA

**Martina Morris**

USA

**Marco Mura**

Canada

**Daniel Murray**

Australia

**Immaculate Nankya**

Uganda

**Mark Nelson**

UK

**Ichiro Nishimura**

USA

**David Nolan**

Australia

**Chidi Nwizu**

USA

**Phillipe Nyambi**

USA

**Catriona Ooi**

Australia

**Catherine Orrell**

South Africa

**C. Padmapriyadarsini**

India

**Alexander Pasternak**

The Netherlands

**Kathy Petoumenos**

Australia

**Chansavath Phetsouphanh**

Australia

**Angie Pinto**

Australia

**Jeffrey Post**

Australia

**Miguel Quinones-Mateu**

USA

**Roshan Ramlal**

USA

**Michael Roche**

Australia

**Jean-Pierre Routy**

Canada

**Allison Ruark**

USA

**Nathan Ryder**

Australia

**Carlos Seas**

Peru

**Leslie Shanks**

Canada

**Pasang Sherpa**

USA

**Tatsuo Shioda**

Japan

**Michael Silverman**

Canada

**Don Smith**

Australia

**Kelli Snyders**

Australia

**Marcelo Soares**

Brazil

**Tim Spelman**

Australia

**Sanjay Swaminathan**

Australia

**Sue Swindells**

USA

**Alice Tang**

USA

**Denis Tebet**

USA

**Janine Trevillyan**

Australia

**Stuart Turville**

Australia

**ECM Van Gorp**

The Netherlands

**Johan Van Griensven**

Belgium

**Guido Vanham**

Belgium

**Angela Wahl**

USA

**Handan Wand**

Australia

**Alan Winston**

UK

**Ian Woolley**

Australia

**H. Manisha Yapa**

Australia

**Evy Yunihastuti**

Indonesia

**Yong-Tang Zheng**

China

**John Ziegler**

Australia

